# Evaluation of Dose-Dependent Efficacy and Mechanisms of Aspirin Combined with Intravenous Immunoglobulin in a Murine Model of Kawasaki Disease Vasculitis

**DOI:** 10.3390/ijms27219783

**Published:** 2026-10-30

**Authors:** Kuang-Jen Chien, Shih-Hui Huang, Wei-De Wang, Chun-Kuei Huang, Ho-Chang Kuo, Yao-Tsung Yeh, Chu-Chuan Lin, Kai-Sheng Hsieh, Ken-Pen Weng

**Affiliations:** 1 Congenital Structural Heart Disease Center, Department of Pediatrics, Kaohsiung Veterans General Hospital, 386 Ta-Chung 1st Road, Kaohsiung 813, Taiwankuei8971138@gmail.com (C.-K.H.);; 2 Department of Nursing, Fooyin University, Kaohsiung 831, Taiwan; 3 Zuoying Armed Forces General Hospital, Kaohsiung 813, Taiwan; 4 Department of Respiratory Therapy, Kaohsiung Chang Gung Memorial Hospital, Kaohsiung 833, Taiwan; 5 Department of Pediatrics and Kawasaki Disease Center, Kaohsiung Chang Gung Memorial Hospital, Kaohsiung 833, Taiwan; 6 College of Medicine, Chang Gung University, Taoyuan 333, Taiwan; 7 Aging and Diseases Prevention Research Center, Department of Medical Laboratory Science and Biotechnology, Fooyin University, Kaohsiung 831, Taiwan; 8 Department of Pediatrics, China Medical University Children’s Hospital, Taichung 404, Taiwan; 9 School of Medicine, National Yang Ming Chiao Tung University, Taipei 112, Taiwan

**Keywords:** Kawasaki disease, LCWE vasculitis, aspirin dosing, intravenous immunoglobulin (IVIG), nuclear factor kappa-B (NF-κB) signaling, matrix metalloproteinance-9 (MMP-9), elastic lamina remodeling

## Abstract

The requirement and molecular contribution of acute high-dose aspirin (ASA) in the treatment of Kawasaki disease (KD) remain clinically controversial. This study aimed to investigate the dose-dependent mechanism and pharmacodynamics of adjunctive ASA in an experimental KD mouse model. Male C57BL/6 mice were injected with Lactobacillus casei cell wall extract (LCWE; 100 µg/day, Days 1–7) to induce systemic coronary arteritis. On Day 14, mice received intravenous immunoglobulin (IVIG) (2 g/kg, i.p.) monotherapy (G3) or IVIG combined with High-dose (100 mg/kg; G4), Medium-dose (50 mg/kg; G5), or Low-dose ASA (5 mg/kg; G6) daily via oral gavage for 3 days (Days 14–16), followed by a 5-day drug washout until sacrifice on Day 21. Coronary vasculitis and internal elastic lamina integrity were quantified using H&E and Verhoeff–Van Gieson (VVG) staining. Heart tissue activation of p-nuclear factor kappa-B (NF-κB) and downstream matrix metalloproteinance-9 (MMP-9) expression were evaluated by Western blot. High-dose ASA combined with IVIG (G4) achieved statistically superior suppression of upstream NF-κB phosphorylation (p-NF-κB/NF-κB ratio) and perivascular leukocyte infiltration compared to IVIG monotherapy (G3) (*p* < 0.05). This acute upstream blockade directly preserved arterial wall architecture, with G4 demonstrating near-baseline structural restoration of the internal elastic lamina on VVG scoring (G4 vs. G3, *p* < 0.05). Following the 5-day drug washout, downstream MMP-9 expression in G4 converged with G3 levels due to pharmacodynamic waning, whereas suboptimal mid/low-dose ASA regimens (G5/G6) displayed lingering MMP-9 elevation, indicating persistent low-grade matrix remodeling. Acute high-dose ASA provides a non-redundant, synergistic anti-inflammatory effect alongside IVIG to prevent early elastolysis. Maintaining adequate acute ASA dose followed by careful post-withdrawal tapering is vital to prevent delayed arterial wall remodeling and coronary aneurysm progression.

## 1. Introduction

Kawasaki disease (KD) is an acute, self-limiting systemic vasculitis that primarily affects infants and young children. It stands as the leading cause of acquired heart disease in children in developed nations, carrying a critical risk of developing coronary artery aneurysms (CAA) if left untreated [1,2,3].

While high-dose intravenous immunoglobulin (IVIG) combined with aspirin remains the standard primary therapy during the acute phase, the therapeutic necessity and efficacy of concurrent high-dose aspirin have become a subject of intense clinical debate. Recent multi-center trials and meta-analyses suggest that high-dose aspirin does not lower the incidence of CAA but significantly correlates with systemic risks such as transient anemia, leading to highly inconsistent outcomes across global treatment guidelines [4,5,6].

However, high-dose aspirin use is associated with a risk of toxicity, such as gastric irritation and Reye’s syndrome, without a clear additional benefit over lower doses. This dosage controversy highlights the need for further investigation into how aspirin doses modulate specific systemic and tissue-level pathways. To systematically bridge this translational gap, precise quantification of coronary arterial dimensions is required; in clinical settings and advanced preclinical imaging, the assessment of coronary artery dimensions relies heavily on standardized Z-scores to effectively mitigate body surface area variations and track localized aneurysmal expansion [7,8]. To fill this research gap, this study used Lactobacillus casei cell wall extract (LCWE) to induce a vasculitis model in C57BL/6 mice, aiming to systematically investigate the effects of different doses of aspirin combined with IVIG on microscopic pathology and molecular signaling pathways. Through this standardized gene platform, we hope to clarify whether high-dose aspirin has potential molecular protective value in the acute phase and provide a mechanistic basis for future “tailor-made” precision treatment strategies, aiming to maximize vascular protection while reducing unnecessary drug risks.

## 2. Results

### 2.1. Physiological Observations and Systemic Assessment

**Figure 1:**
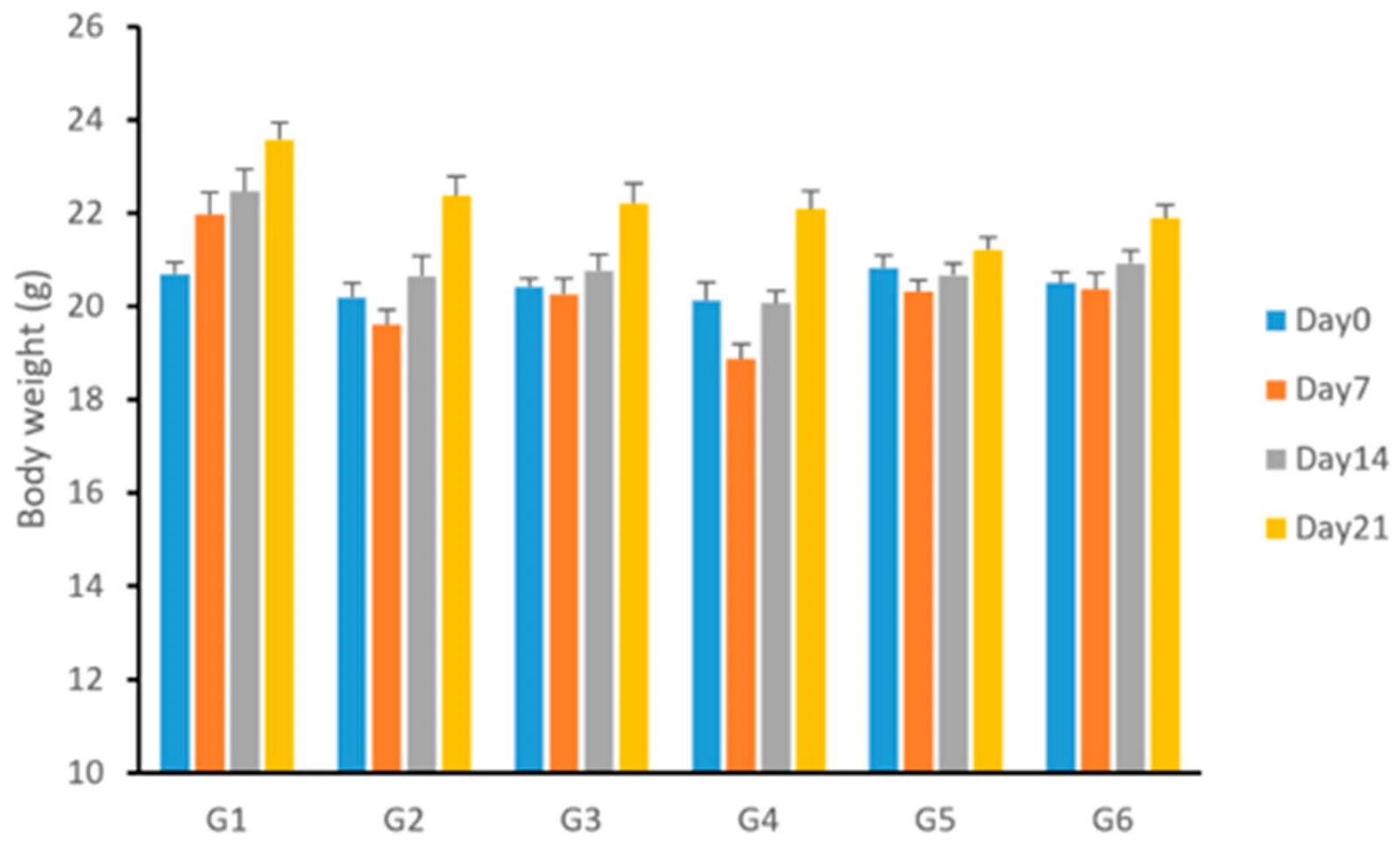
Changes in body weight during the 21-day experimental period across experimental groups (*n* = 6 per group). Data depict body weight trajectories measured on Days 0, 7, 14, and 21. Transient weight reduction was observed on Day 7 following the cessation of LCWE induction across all disease groups (with the exception of G1 Control), followed by progressive recovery through Day 21. Experimental groups: G1 (Control), G2 (KD only), G3 (KD + IVIG), G4 (KD + IVIG + High ASA, 100 mg/kg), G5 (KD + IVIG + Medium ASA, 50 mg/kg), and G6 (KD + IVIG + Low ASA, 5 mg/kg). Data are presented as mean ± SEM.

To monitor the systemic effect of LCWE induction, body weight was measured on Days 0, 7, 14, and 21 (Figure 1). Following seven consecutive days of LCWE administration (Day 1 to Day 7), all LCWE-treated groups (G2–G6) exhibited varying degrees of body weight reduction on Day 7 compared to their baseline levels on Day 0 (ranging from −0.5% to −6.0% weight loss across groups), whereas the control group (G1) showed normal developmental weight gain (+6.3% increase).

Following the cessation of LCWE injection, a steady recovery in body weight was observed across all LCWE-treated groups from Day 14 through Day 21, as illustrated in Figure 1. This physiological trend indicates that the animals successfully survived the acute phase of systemic inflammation induced by LCWE challenge.

### 2.2. Assessment of Cardiac Hypertrophy

To evaluate potential macroscopic structural changes, the heart weight-to-body weight (HW/BW) ratio was calculated at the study endpoint (Day 21). Although the G2 (KD only) group exhibited a slightly elevated HW/BW ratio (0.00552 ± 0.00067) compared to the G1 control group (0.00513 ± 0.00021), one-way ANOVA revealed no statistically significant differences among any of the experimental groups at this time point (*p* > 0.05). 

This absence of overt cardiac hypertrophy suggests that the acute systemic inflammation induced by LCWE had not yet led to gross cardiac structural alterations by Day 21. These findings underscore the critical necessity of shifting our focus from macroscopic physiological indices to microscopic vascular histopathology to precisely characterize the protective efficacy of the treatments on coronary integrity. 

### 2.3. Histopathological Evaluation of Coronary Vasculitis and Elastic Lamina Damage

To characterize microvascular pathology, coronary artery sections were evaluated using Hematoxylin and Eosin (H&E) staining for perivascular inflammation and Verhoeff–Van Gieson (VVG) staining for internal elastic lamina integrity (*n* = 6 per group). 

**Figure 2:**
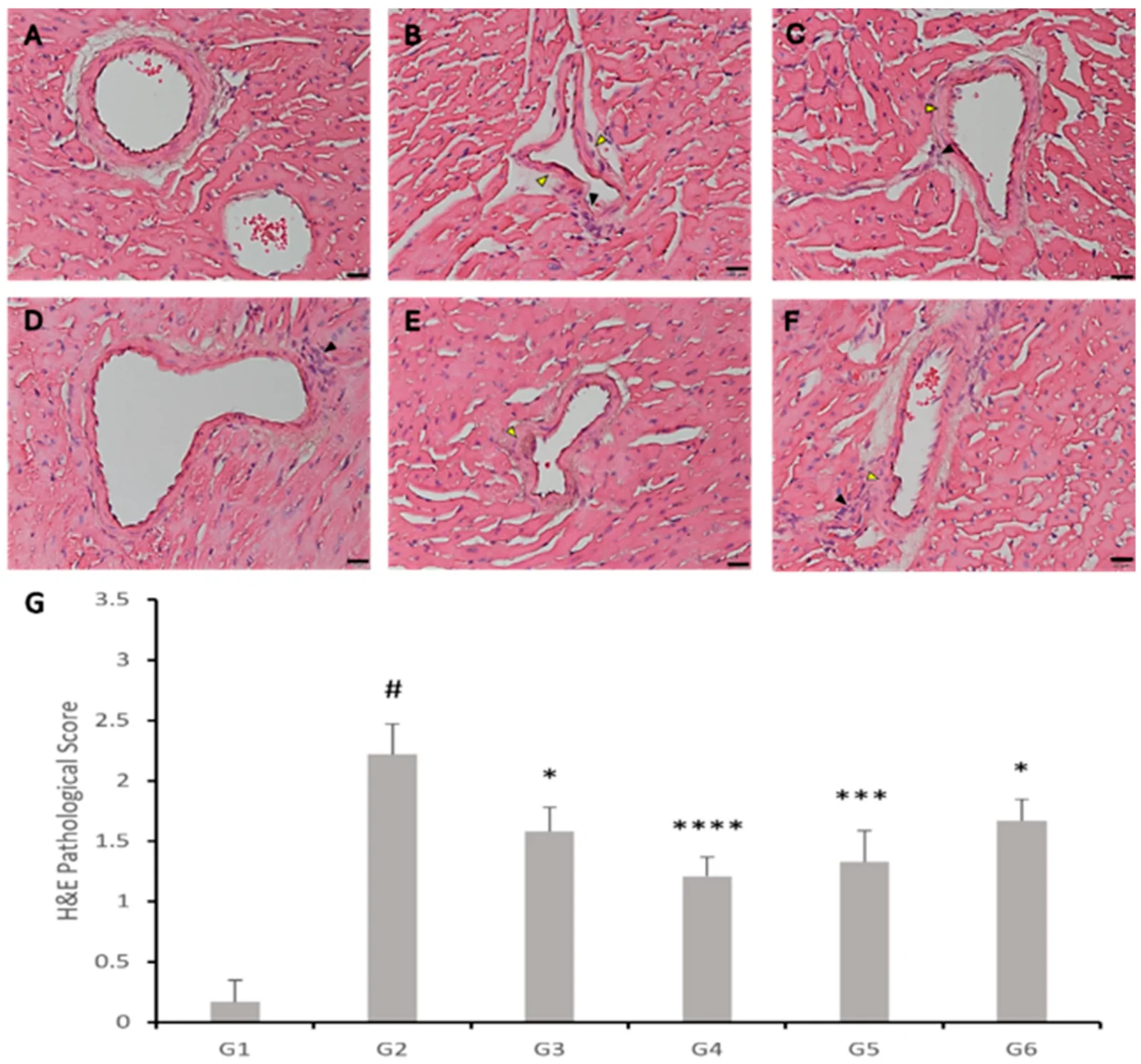
Histopathological evaluation and pathological scoring of coronary perivascular inflammation by H&E staining. (**A**–**F**) Representative histopathological images of coronary arteries stained with Hematoxylin and Eosin (H&E) from each group: (**A**) Normal control (G1), (**B**) KD + Placebo (G2), (**C**) KD + IVIG (G3), (**D**) KD + IVIG + High-dose Aspirin (100 mg/kg; G4), (**E**) KD + IVIG + Medium-dose Aspirin (50 mg/kg; G5), and (**F**) KD + IVIG + Low-dose Aspirin (5 mg/kg; G6). Yellow arrowheads indicate medial disruption and architectural disorganization; black arrowheads highlight perivascular leukocyte and inflammatory cell infiltration. Scale bar = 20 μm. (**G**) Quantitative evaluation of H&E pathological scores across experimental groups. Data are presented as mean ± SD (*n* = 6 mice per group, evaluated from non-consecutive step sections per animal). Statistical significance was calculated using the Kruskal–Wallis test followed by Dunn’s post hoc test. # *p* < 0.001 vs. G1; * *p* < 0.05, *** *p* < 0.001, **** *p* < 0.0001 vs. G2. (Note: Combination therapy with high-dose aspirin showed superior anti-inflammatory efficacy compared to IVIG monotherapy, G4 vs. G3, *p* < 0.05).

In H&E analysis, LCWE administration induced extensive perivascular leukocyte infiltration and tissue disorganization, markedly increasing H&E histopathological scores compared to normal controls (G2 vs. G1, *p* < 0.001) (Figure 2A–G). IVIG monotherapy significantly attenuated inflammatory cell infiltration relative to the untreated KD placebo group (G3 vs. G2, *p* < 0.05). Combination therapies with IVIG and aspirin further reduced pathological severity across all dosage levels (G4, G5, and G6 vs. G2, all *p* < 0.05). Notably, the high-dose aspirin combination group demonstrated the most pronounced anti-inflammatory effect (G4 vs. G2, *p* < 0.0001). Furthermore, adjunctive high-dose aspirin achieved significantly superior suppression of vascular inflammation compared to IVIG monotherapy alone (G4 vs. G3, *p* < 0.05) (Figure 2G). 

**Figure 3:**
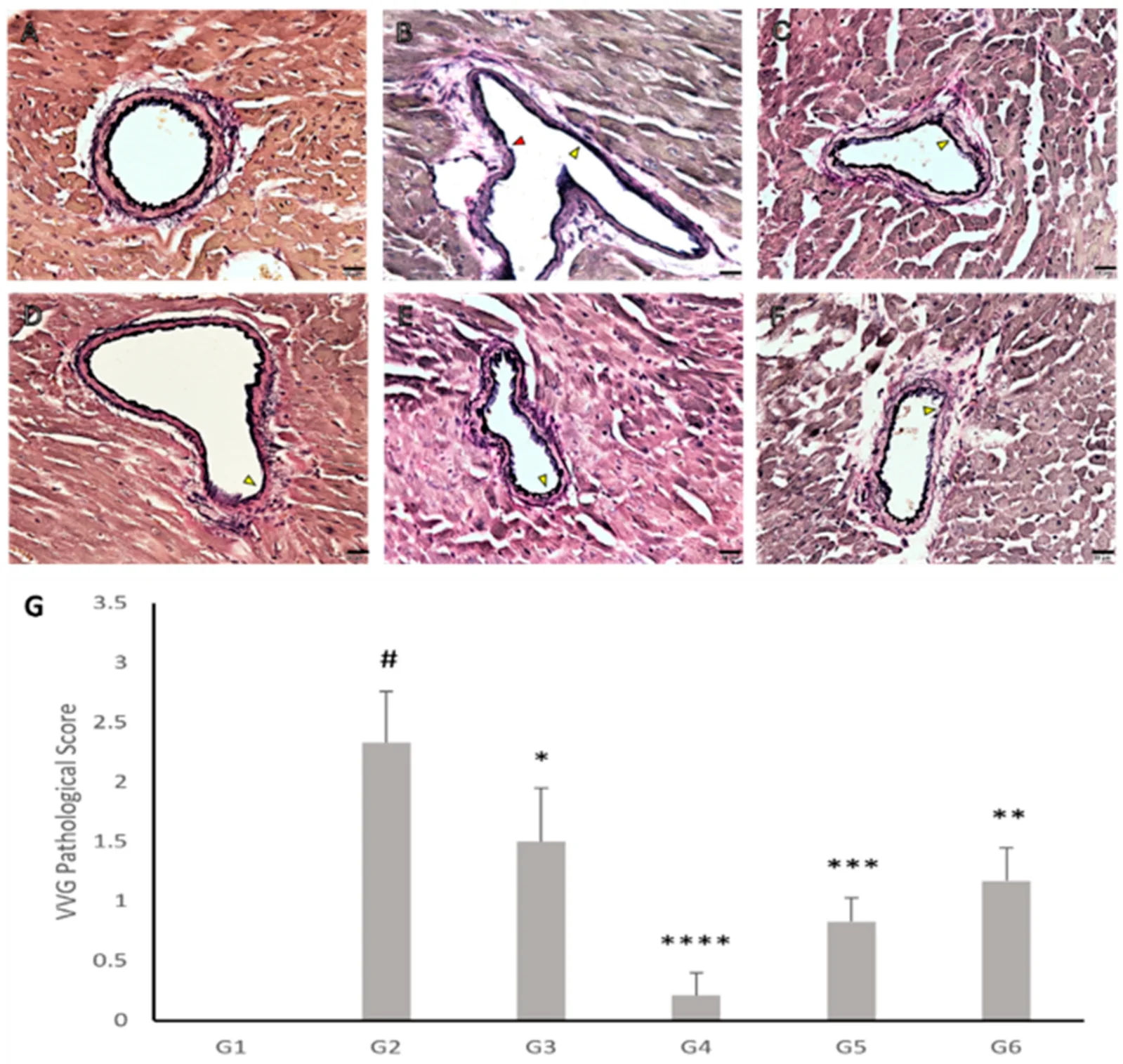
Histopathological evaluation and pathological scoring of coronary arterial elastic lamina integrity by VVG staining. (**A**–**F**) Representative Verhoeff–Van Gieson (VVG) stained coronary artery sections from each group: (**A**) Normal control (G1), (**B**) KD + Placebo (G2), (**C**) KD + IVIG (G3), (**D**) KD + IVIG + High-dose Aspirin (100 mg/kg; G4), (**E**) KD + IVIG + Medium-dose Aspirin (50 mg/kg; G5), and (**F**) KD + IVIG + Low-dose Aspirin (5 mg/kg; G6). Black/purple wavy bands denote intact internal elastic lamina; red arrowheads represent severe elastic lamina ablation or disruption, and yellow arrowheads indicate elastic lamina stretching and attenuation. Scale bar = 20 μm. (**G**) Quantitative evaluation of VVG pathological scores across experimental groups. Data are presented as mean ± SD (*n* = 6 mice per group). Statistical significance was determined by Kruskal–Wallis test followed by Dunn’s post hoc test. # *p* < 0.001 vs. G1; * *p* < 0.05, ** *p* < 0.01, *** *p* < 0.001, **** *p* < 0.0001 vs. G2. (Note: Combination therapy with high-dose aspirin showed significantly enhanced protective efficacy compared with IVIG monotherapy, G4 vs. G3, *p* < 0.05).

In VVG structural assessment, LCWE-induced KD model mice displayed severe elastolysis and structural disruptions of the internal elastic lamina, yielding significantly elevated VVG pathological scores (G2 vs. G1, *p* < 0.001) (Figure 3A–G). IVIG monotherapy significantly reduced elastic lamina degradation compared to the G2 group (G3 vs. G2, *p* < 0.05). Adjunctive aspirin administration further protected elastic fiber integrity in a dose-dependent manner (G4, G5, and G6 vs. G2, all *p* < 0.05). Importantly, high-dose aspirin combination therapy showed exceptional structural preservation, restoring pathological scores near baseline levels (G4 vs. G2, *p* < 0.0001). Consistent with H&E findings, high-dose aspirin combined with IVIG offered significantly greater elastic lamina protection than IVIG monotherapy (G4 vs. G3, *p* < 0.05) (Figure 3G). 

### 2.4. Molecular Signaling and Proteolytic Markers Analysis

To elucidate the molecular mechanisms underlying the observed vascular protection, Western blot analysis was performed to evaluate the activation of the central inflammatory transcription factor Nuclear factor kappa-B (NF-kB) and the expression of the matrix-degrading enzyme MMP-9 in heart tissues. The activation status of NF-kB p65 was quantified by determining the ratio of phosphorylated NF-kB p65 (p-NF-kB p65) to total NF-kB p65, with GAPDH serving as the internal loading control for normalization.

#### High-Dose Aspirin Combined with IVIG Suppresses NF-kB Activation and MMP-9 Expression

**Figure 4:**
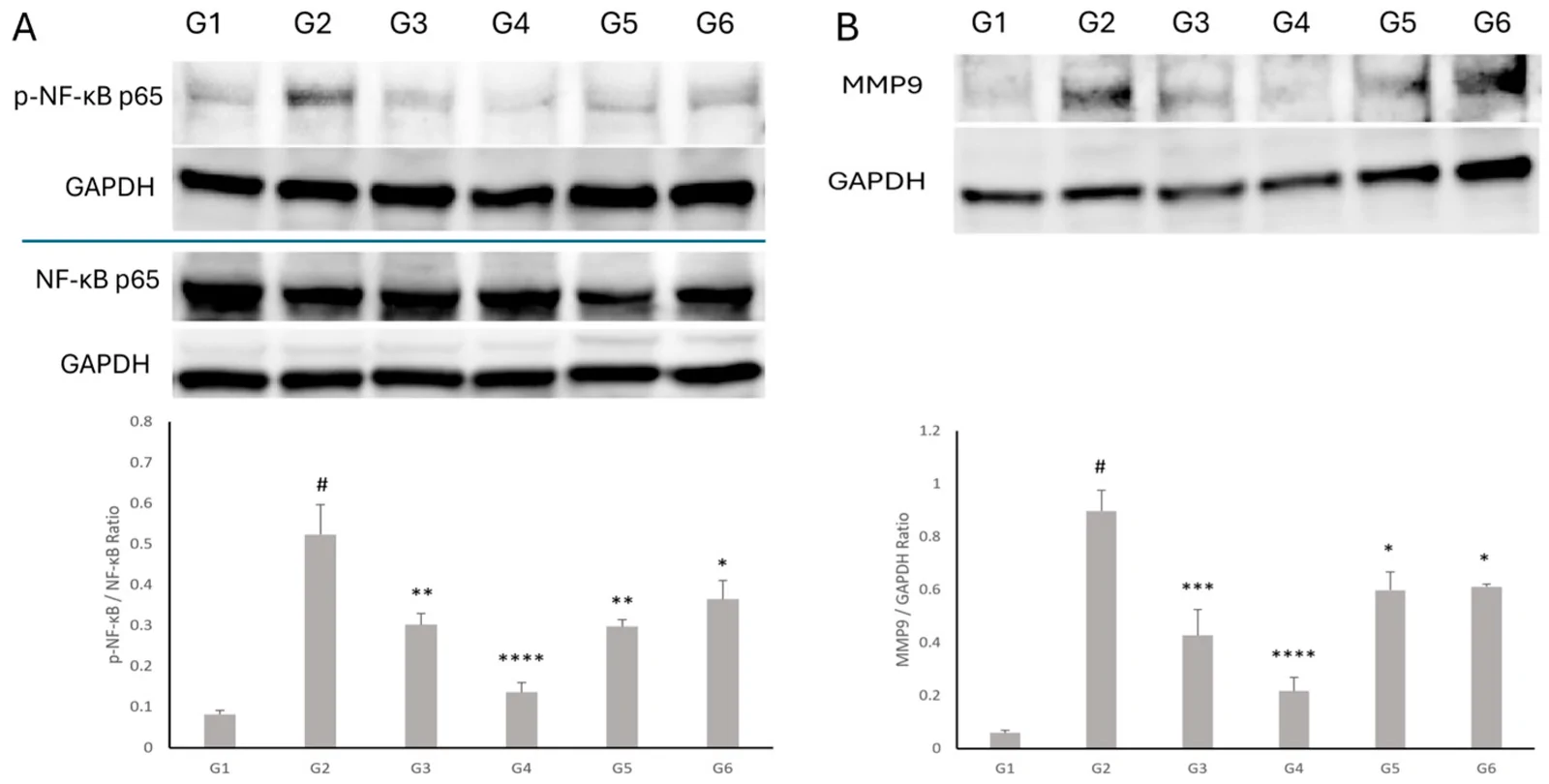
Effects of IVIG and aspirin combination therapy on NF-kB phosphorylation and MMP-9 expression levels in heart tissues. (**A**) Representative Western blot images and quantitative analysis of p-NF-kB p65 and total NF-kB p65 protein ratio. Target proteins were electrophoresed on separate gels, and each signal was normalized to its corresponding internal control GAPDH from the respective blot prior to calculating the p-NF-kB/NF-kB ratio. (**B**) Representative Western blot images and quantitative analysis of MMP-9 protein levels normalized to GAPDH. Data are presented as mean ± SEM. Statistical significance was determined using one-way ANOVA followed by Tukey’s post hoc test. # *p* < 0.0001 vs. G1; * *p* < 0.05, ** *p* < 0.01, *** *p* < 0.001, **** *p* < 0.0001 vs. G2. (Note: Combination therapy with high-dose aspirin significantly enhanced the suppression of NF-kB phosphorylation compared to IVIG monotherapy, G4 vs. G3, *p* < 0.05).

LCWE administration induced robust NF-kB activation, markedly increasing the p-NF-kB p65/NF-kB p65 ratio compared to the normal control group (G2 vs. G1, *p* < 0.0001) (Figure 4A). Treatment with IVIG alone significantly attenuated NF-kB phosphorylation relative to the untreated KD placebo group (G3 vs. G2, *p* < 0.01) (Figure 4A). Combination therapies with IVIG and aspirin further suppressed NF-kB activation across all dosage levels (G4, G5, and G6 vs. G2, all *p* < 0.05) (Figure 4A). Notably, high-dose aspirin adjunctive therapy exhibited the strongest inhibitory effect, restoring p-NF-kB p65 levels near baseline (G4 vs. G2, *p* < 0.0001) (Figure 4A). Furthermore, adjunctive high-dose aspirin achieved significantly greater suppression of NF-kB activation compared to IVIG monotherapy alone (G4 vs. G3, *p* < 0.05) (Figure 4A). 

Consistent with NF-kB activation, heart tissue MMP-9 protein expression was markedly elevated following LCWE induction (G2 vs. G1, *p* < 0.0001) (Figure 4B). IVIG monotherapy significantly reduced MMP-9 expression levels compared to the untreated model group (G3 vs. G2, *p* < 0.001) (Figure 4B). All combination therapy groups also demonstrated significant suppression of MMP-9 levels compared to placebo (G4, G5, and G6 vs. G2, all *p* < 0.05) (Figure 4B). High-dose aspirin adjunctive therapy demonstrated the most pronounced suppression of MMP-9 expression (G4 vs. G2, *p* < 0.0001), bringing MMP-9 expression levels back to levels comparable to the normal control group (G4 vs. G1, *p* = 0.4632) (Figure 4B). 

## 3. Discussion

### 3.1. Differential Efficacy of Combination Therapy on Inflammatory Signals and Histopathology

The management of acute vasculitis in Kawasaki disease (KD) relies heavily on high-dose intravenous immunoglobulin (IVIG) combined with aspirin (ASA) to prevent coronary artery complications [1]. However, the precise synergistic mechanisms of varied ASA dosing regimens on vascular inflammatory pathways and structural integrity remain to be fully characterized. In the present study, at the primary endpoint (Day 21), animals receiving high-dose ASA combined with IVIG (G4) demonstrated superior anti-inflammatory and vascular protective outcomes compared to those receiving IVIG monotherapy (G3) or IVIG with mid- or low-dose ASA (G5 and G6). 

#### 3.1.1. Significant Superiority of High-Dose ASA in Upstream Signaling and Histopathology

A pivotal finding of our study is that the combination of high-dose ASA with IVIG (G4) achieved a significantly greater reduction in the p-NF-kB/NF-kB ratio compared to IVIG monotherapy (G3, *p* < 0.05). This pronounced suppression of NF-kB phosphorylation indicates that acute-phase (Days 14–16) administration of high-dose ASA exerts a robust and sustained inhibitory effect on the activation of this master upstream inflammatory regulator. 

This molecular suppression directly correlated with our histopathological observations [9]. Semi-quantitative scoring of hematoxylin and eosin (H&E)-stained coronary artery sections revealed that perivascular leukocyte infiltration was significantly lower in the G4 group than in the G3 group (*p* < 0.05). Although IVIG monotherapy attenuated overall tissue inflammation, residual inflammatory cell clusters persisted in the perivascular space. The addition of high-dose ASA effectively accelerated the resolution of local inflammation, demonstrating that high-dose ASA is essential during the acute phase to suppress the recruitment and retention of circulating immune cells within the adventitia and perivascular tissues. 

#### 3.1.2. Restoration of Structural Elastic Lamina

Beyond dampening acute cellular infiltration, the preservation of the structural integrity of the coronary vessel wall represents a critical determinant of long-term outcome in KD [10]. Histological evaluation of the internal and external elastic laminae demonstrated distinct structural protection across treatment arms. While all IVIG-containing regimens (G3–G6) provided a baseline level of structural preservation compared to the untreated model group (G2), the G4 group exhibited the most complete and smooth elastic lamina architecture, with minimal fragmentation. 

This structural preservation is highly consistent with the observed suppression of upstream p-NF-kB signaling in G4. By rapidly curtailing NF-kB activation during the critical acute window (Days 14–16), high-dose ASA effectively shielded the vascular extracellular matrix (ECM) from enzymatic attack. Consequently, the combination of high-dose ASA and IVIG prevents the structural weakness of the arterial wall that precedes elastolysis, thereby maintaining coronary architectural integrity. Taken together, our systematic comparison across diverse aspirin dosages highlights a clear dose-dependent threshold. While medium- and low-dose ASA regimens provide partial protection, only the high-dose combination regimen (G4) achieves the necessary spatiotemporal suppression of upstream NF-κB signaling to fundamentally preserve internal elastic lamina architecture and mitigate subsequent matrix remodeling. Furthermore, characterization of post-withdrawal MMP-9 dynamics suggests a theoretical molecular window for therapy transition—bounded by complete upstream p-NF-κB suppression and the onset of compensatory MMP-9 rebound. Referencing established clinical treatment concepts, these mechanistic benchmarks provide a strong rationale for designing future animal studies to explicitly evaluate whether seamless step-down bridging within this specific window can eliminate pharmacodynamic gaps and optimize long-term vascular preservation.

### 3.2. Temporal Pharmacodynamics and the MMP9 Trajectory (Day 17–21 Washout Impact)

#### 3.2.1. Waning Effect of Aspirin on Downstream MMP9 Expression

A critical aspect of our experimental design is the temporal sequence of drug administration and tissue sampling. Animals received therapy during the acute inflammatory window (Days 14–16), which was followed by a 5-day drug withdrawal period prior to terminal sacrifice at Day 21. While high-dose aspirin combined with IVIG (G4) exhibited robust suppression of upstream p-NF-kB signaling, its inhibitory effect on downstream matrix metalloproteinase-9 (MMP-9) expression diminished by Day 21, showing no statistically significant difference compared to IVIG monotherapy (G3). 

This convergence between G4 and G3 can be explained by pharmacodynamic waning following drug cessation [11]. Aspirin functions primarily as an irreversible inhibitor of cyclooxygenase-1 (COX-1) with transient, dose-dependent anti-inflammatory kinetics [12]. Because MMP-9 is a downstream effector enzyme driven by sustained transcription factors and local cellular remodeling, its protein level reflects a delayed kinetic profile relative to upstream signaling events like NF-kB phosphorylation [13]. Upon the cessation of high-dose aspirin therapy at Day 16, the direct pharmacological pressure suppressing downstream transcriptional activity gradually dissipated over the 5-day washout period [11]. By Day 21, the observed MMP-9 levels in the G4 group predominantly reflected the lingering, steady-state immune-modulatory influence of IVIG rather than active aspirin suppression [13].

#### 3.2.2. Compensatory Dynamics in Mid/Low-Dose Regimens

Intriguingly, animals receiving mid-dose (G5) or low-dose (G6) aspirin in combination with IVIG exhibited sustained or slightly elevated MMP-9 expression levels at Day 21 compared to the high-dose group (G4). Rather than indicating a therapeutic failure, this observation reflects the intricate balance between incomplete acute-phase inflammatory suppression and post-withdrawal compensatory tissue remodeling [13].

When aspirin is administered at suboptimal anti-inflammatory dosages (mid or low doses), it fails to fully abolish the acute activation of upstream transcriptional regulators such as NF-kB during Days 14–16 [14]. Consequently, low-grade, persistent vascular inflammation continues to drive the transcription of downstream effector genes, including MMP-9 [13]. Furthermore, suboptimal suppression of cyclooxygenase pathways can induce an arachidonic acid metabolic shunt, diverting enzymatic flux toward pro-inflammatory leukotriene production or modulating tissue inhibitors of metalloproteinases (TIMPs), which in turn stabilizes or upregulates active MMP-9 [15,16]. Following the 5-day drug washout period (Days 17–21), this persistent low-level inflammatory drive manifests as a compensatory increase in MMP-9, reflecting active extracellular matrix degradation and reactive vascular remodeling in response to unmitigated tissue injury [17]. Therefore, insufficient acute dosing not only compromises immediate anti-inflammatory efficacy but also permits delayed matrix remodelling dynamics following treatment withdrawal [13].

### 3.3. Clinical Translation and Dosing Strategy

#### 3.3.1. Acute Phase High-Dose ASA Requirement for Inflammatory Arrest

The clinical management of Kawasaki disease (KD) during the acute phase has long debated the necessity and exact anti-inflammatory contribution of high-dose aspirin when administered alongside intravenous immunoglobulin (IVIG) [1]. Our experimental findings provide convincing, objective evidence supporting the mandatory inclusion of high-dose aspirin during the early critical window. By demonstrating that the G4 combination regimen (high-dose ASA + IVIG) achieved a statistically significant reduction in both the p-NF-kB/NF-kB activation ratio and perivascular leukocyte infiltration compared to IVIG monotherapy (G3, *p* < 0.05), our data indicate that high-dose aspirin provides a non-redundant, potent synergistic anti-inflammatory effect. 

While IVIG acts as a broad-spectrum immunomodulatory agent, high-dose aspirin rapidly establishes acute tissue-level blockade against upstream transcriptional activation [14,18]. This dual-action approach is clinically crucial because rapid arrest of acute perivascular inflammatory cell recruitment is required before irreversible arterial destruction takes place [19]. Consequently, reducing or omitting aspirin during the hyper-inflammatory acute phase may compromise early inflammatory clearance within the adventitia, leaving the vessel wall susceptible to ongoing microvascular injury [1].

#### 3.3.2. Implications for Drug Tapering and Long-Term CAA Prevention

While acute high-dose aspirin is required to arrest hyper-acute tissue inflammation, our temporal observation of MMP-9 expression at Day 21 highlights the importance of optimizing the drug tapering schedule. In clinical practice, high-dose aspirin is typically tapered to a low anti-platelet dose once the patient remains afebrile for 48–72 h [1]. However, our findings show that following drug cessation, downstream matrix remodelling markers like MMP-9 can exhibit delayed, persistent expression or mild post-withdrawal rebound [13].

This lag between upstream signaling inhibition (e.g., p-NF-kB) and downstream tissue matrix stabilization suggests that subclinical arterial wall remodeling may continue even after systemic inflammatory parameters (such as fever or CRP) have normalized [20]. Therefore, abrupt discontinuation or overly rapid tapering of aspirin without monitoring downstream remodeling dynamics could leave a transient window of vulnerability where active matrix degradation proceeds uninhibited [21,22]. Clinically, maintaining low-dose anti-platelet aspirin not only prevents microthrombus formation but may also support long-term vascular lumen stability while the extracellular matrix fully restores [1,22]. Future therapeutic strategies may benefit from combining acute anti-inflammatory blockade with prolonged targeted matrix protection to minimize long-term CAA progression. Taken together, our experimental findings provide a mechanistically sound rationale supporting a balanced two-stage therapeutic concept in Kawasaki disease management: initial high-dose ASA combined with IVIG during the acute window to rapidly quench upstream transcriptional signaling, followed by a controlled step-down to low-dose maintenance to mitigate post-withdrawal matrix remodeling rebounds and favor long-term vascular stability.

### 3.4. Study Limitations and Future Directions

Despite providing valuable insights into the synergistic mechanism and anti-inflammatory kinetics of aspirin combined with IVIG in the LCWE-induced Kawasaki disease vasculitis model, several potential limitations of this study should be acknowledged.

First, our tissue evaluations were restricted to fixed key time points—specifically Day 21 terminal sacrifice following a 5-day washout period (Days 17–21). While this temporal framework successfully captured the post-acute matrix remodeling status and delayed MMP-9 expression dynamics, it did not include continuous intermediate tissue harvesting during the active treatment phase (Days 14–16). Consequently, the real-time, peak drug kinetics of NF-kB inhibition during active dosing were inferred relative to terminal sacrifice data rather than direct acute-phase serial histopathology [13].

Second, although the LCWE murine model is widely accepted as the gold standard animal model for mimicking human Kawasaki disease arteritis [23], murine vascular physiology exhibits inherently higher baseline endothelial regenerative capacity compared to humans. Furthermore, translating murine dosing kinetics to pediatric clinical practice remains challenging due to the highly variable serum salicylic acid pharmacokinetics observed in children with acute Kawasaki disease [24].

Finally, our protein-level analyses focused primarily on p-NF-kB and total MMP-9 expression. While MMP-9 serves as a reliable marker for matrix degradation, future investigations incorporating zymography for enzymatic activity assays, together with comprehensive profiling of tissue inhibitors of metalloproteinases (TIMPs) and other matrix metalloproteinase isoforms (e.g., MMP-2, MMP-12), will provide a more granular understanding of extracellular matrix balance [17]. Addressing these questions in future studies with multi-time point longitudinal sampling will further refine the precise therapeutic window for combination regimens in KD. Several limitations of the present study warrant consideration. First, our experimental design was intentionally focused on the standard clinical regimen of adjunctive aspirin combined with frontline IVIG to reflect real-world therapeutic management, and thus an aspirin-alone group was not included. Second, our mechanistic investigation was centered on the canonical p-NF-κB/MMP-9 axis; however, vascular remodeling in Kawasaki disease is inherently complex. Future studies incorporating broader transcriptomic or proteomic profiling to investigate other inflammatory signaling pathways and systemic vascular beds will be valuable to further delineate the complete regulatory network. Third, while the LCWE murine model provides a highly reliable platform for acute coronary arteritis, intrinsic species-specific variations in metabolic clearance rates and acute phase reactants necessitate caution when extrapolating these findings directly to clinical practice. Finally, future investigations employing longitudinal echocardiographic functional evaluations, multi-time-point temporal evaluations and direct experimental testing of high-to-low dose step-down maintenance regimens will be essential to clarify optimal transition timing and further refine these findings.

## 4. Materials and Methods

### 4.1. Animal Ethics and Housing

All animal experiments were conducted in accordance with the legislation on the protection of animals and were approved by the Institutional Animal Care and Use Committee (IACUC) of Kaohsiung Veterans General Hospital (Protocol No. IACUC-2503-2802-25007-VGHKS). Five-week-old male C57BL/6 mice were used in this study, purchased from the National Laboratory Animal Center, Taiwan. Mice were housed in a pathogen-free facility at 22 ± 2 °C with a 12 h light/dark cycle and provided with sterile food and water ad libitum. 

### 4.2. Preparation of LCWE

Lactobacillus casei (ATCC 11578) cell wall extract (LCWE) was prepared according to established protocols. Briefly, L. casei cells were cultured in Lactobacillus MRS broth and harvested after reaching the stationary phase. The cells were treated with 4% SDS overnight, followed by incubation with RNase, DNase I, and trypsin to remove non-cell wall components. The final pellet was sonicated and centrifuged to obtain the supernatant. The concentration of the LCWE was determined based on its rhamnose content using a phenol-sulfuric acid colorimetric assay [23,25,26,27].

### 4.3. KD Model Induction and Experimental Groups

**Figure 5:**
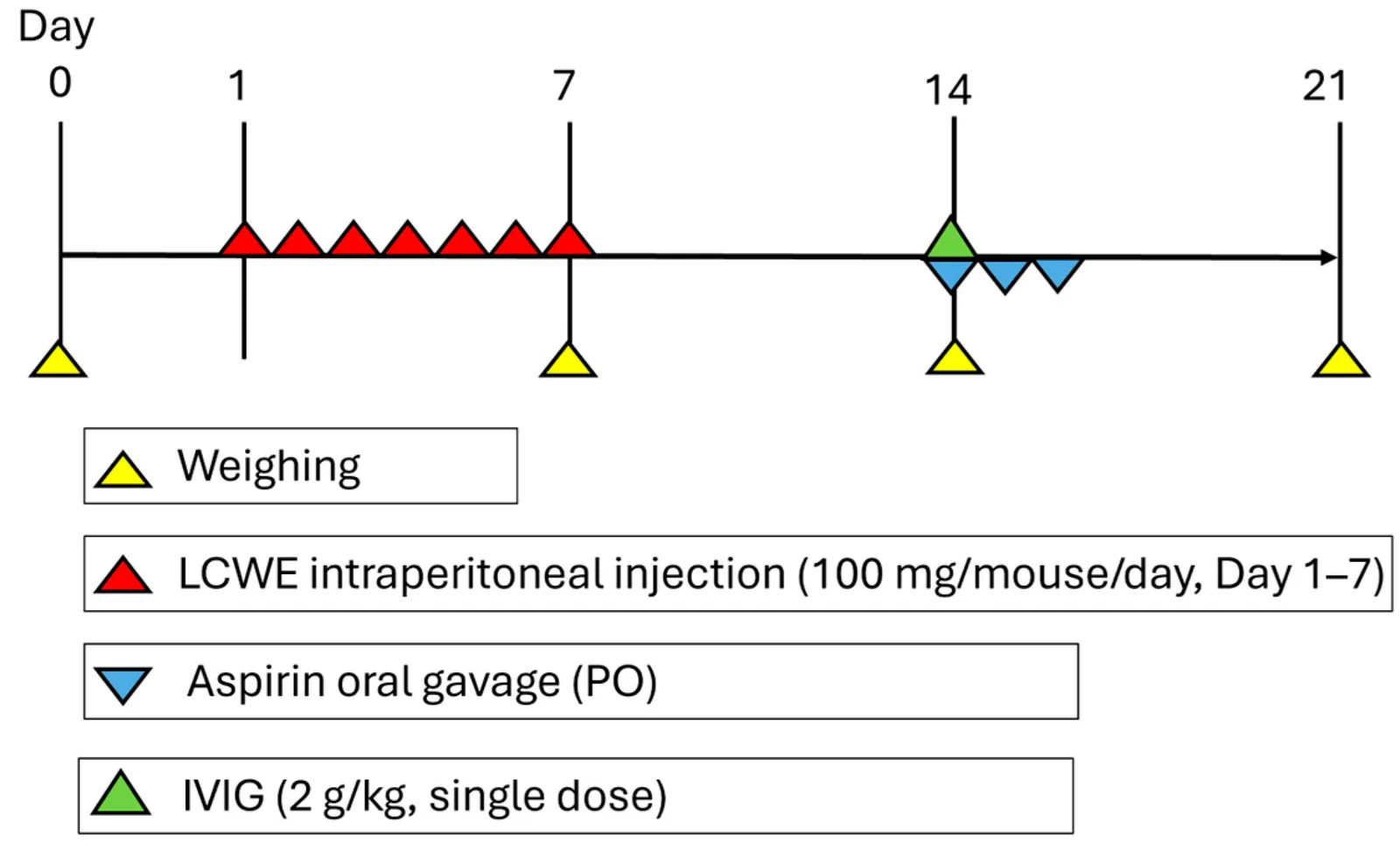
Experimental schematic and timeline of LCWE-induced Kawasaki disease (KD) vasculitis and treatment. (1) Induction: LCWE (100 μg/mouse/day, i.p.) was administered daily from Day 1 to Day 7; (2) Intervention: A single dose of IVIG (2 g/kg, i.p.) was administered on Day 14 post-induction; (3) Treatment: Aspirin (P.O.) was administered daily from Day 14 to Day 16; (4) Endpoint: Animals were sacrificed on Day 21 for comprehensive pathological and molecular analysis. Body weight was monitored on Days 0, 7, 14, and 21 to evaluate systemic health status.

To induce Kawasaki disease (KD) vasculitis, five-week-old male C57BL/6 mice were intraperitoneally (i.p.) injected with LCWE at a dose of 100 μg/mouse/day for seven consecutive days (Day 1 to Day 7). The detailed experimental procedure and treatment timeline are illustrated in Figure 5. Mice were randomly assigned into six experimental groups (*n* = 6 per group):

- G1 (Control): PBS injection + Saline placebo.
- G2 (KD only): LCWE induction + Placebo.
- G3 (KD + IVIG): LCWE induction + IVIG (2 g/kg, i.p. on Day 14).
- G4 (High ASA): LCWE + IVIG + Aspirin (100 mg/kg/day, P.O. from Day 14 to Day 16).
- G5 (Medium ASA): LCWE + IVIG + Aspirin (50 mg/kg/day, P.O. from Day 14 to Day 16).
- G6 (Low ASA): LCWE + IVIG + Aspirin (5 mg/kg/day, P.O. from Day 14 to Day 16).

### 4.4. Drug Administration

Standard intravenous immunoglobulin (IVIG) was administered as a single dose of 2 g/kg on Day 14 post-induction via intraperitoneal injection. Aspirin (Aspirin Protect; Bayer, Leverkusen, Germany) was administered via oral gavage (P.O.) once daily for three consecutive days (Day 14 to Day 16) following IVIG administration to simulate clinical combination therapy. Control and KD-only groups received volume-matched normal saline via the corresponding routes. 

### 4.5. Physiological, Histopathological, and Statistical Assessment

Body weight was recorded on Days 0, 7, 14, and 21 to evaluate systemic inflammatory wasting. On Day 21, mice were humanely euthanized, and heart tissues were harvested and weighed. The heart weight-to-body weight ratio (HW/BW) was calculated as a surrogate marker for cardiac hypertrophy. 

For histopathological evaluations, heart tissues containing the aortic root and coronary arteries were fixed in 10% neutral buffered formalin, embedded in paraffin, and cut into 5 μm thick sections. Step sectioning was performed by collecting 2 to 4 non-consecutive sections separated by 100 μm intervals (discarding 20 intervening 5 μm sections) for each animal (*n* = 6 per group). The quantitative scores from multiple sections per mouse were averaged to represent a single biological replicate for statistical analysis. 

Hematoxylin and Eosin (H&E) staining was performed to evaluate perivascular inflammation. Perivascular inflammatory infiltration was semi-quantitatively scored on a scale of 0 to 4: Score 0, no inflammatory cell infiltration; Score 1, minimal/scattered single inflammatory cells (1–4 cells); Score 2, mild/small focal inflammatory cell clusters; Score 3, moderate/multifocal or significant intramural infiltration; Score 4, severe/diffuse circumferential or semi-circumferential cuffing infiltration. 

Verhoeff–Van Gieson (VVG) staining (Biotna, Catalog No. TASS11) was conducted to evaluate internal elastic lamina integrity and vascular remodeling according to the manufacturer’s protocol. Briefly, hydrated sections were stained in Verhoeff’s working solution for 20 min, differentiated in 2% ferric chloride solution for 1 to 2 min, treated with 5% sodium thiosulfate for 1 min, and counterstained with Van Gieson’s solution for 3 min. Elastic fibers were visualized as dark blue to black. Elastic lamina damage and remodeling were semi-quantitatively scored on a scale of 0 to 3: Score 0, intact 360-degree continuity with sharp waviness; Score 1, mild remodeling, duplication, or local flattening without disruption; Score 2, focal disruption or elastolysis involving less than 25% of the vessel circumference; Score 3, severe elastolysis or multifocal disruptions involving more than 25% of the vessel circumference [27,28,29]. 

Continuous parametric data (HW/BW ratio and body weight changes) were analyzed using one-way analysis of variance (ANOVA) followed by Tukey’s post hoc test. Semi-quantitative histopathological scores (H&E and VVG scores) were analyzed using the non-parametric Kruskal–Wallis test followed by Dunn’s post hoc test. Histopathological and physiological data are expressed as mean ± SD, and a *p*-value < 0.05 was considered statistically significant.

### 4.6. Western Blot Analysis

Total proteins were extracted from heart tissues using RIPA lysis buffer supplemented with protease and phosphatase inhibitors. Protein concentrations were determined using a BCA protein assay kit. Equal amounts of protein samples (40 μg) were separated by SDS-PAGE and subsequently transferred onto PVDF membranes. After blocking with 5% non-fat milk for 1 h at room temperature, the membranes were incubated overnight at 4 degrees C with primary antibodies against NF-kB p65 (1:1000, GeneTex, Cat No. GTX102090), p-NF-kB p65 (1:1000, GeneTex, Cat No. GTX133899), MMP-9 (1:1000, GeneTex, Cat No. GTX100458), and GAPDH (1:5000, GeneTex, Cat No. GTX100118).

Following incubation with appropriate HRP-conjugated secondary antibodies, protein bands were visualized using an enhanced chemiluminescence (ECL) detection system. Densitometric analysis of the bands was performed using Image software (version 1.54; National Institutes of Health, Bethesda, MD, USA). Target protein intensities were normalized to GAPDH as an internal loading control to calculate relative protein expression levels. Quantitative data are presented as mean ± SEM. Statistical comparisons were performed using one-way ANOVA followed by Tukey’s post hoc test, and a *p*-value < 0.05 was considered statistically significant. 

## 5. Conclusions

In conclusion, this study provides key mechanistic and pharmacodynamic evidence supporting the mandatory therapeutic role of high-dose aspirin (ASA) combined with IVIG during the acute phase of Kawasaki disease (KD) vasculitis. By utilizing the LCWE-induced murine model, we demonstrated that the addition of high-dose ASA to IVIG (G4) achieves statistically significant superiority over IVIG monotherapy (G3) in suppressing upstream p-NF-kB/NF-kB activation and reducing perivascular leukocyte infiltration (*p* < 0.05). This rapid, synergistic inhibition during the early critical window directly translated into the optimal preservation of arterial structural integrity and internal/external elastic lamina continuity.

Furthermore, our longitudinal observation at Day 21 revealed a pharmacodynamic waning effect on downstream MMP-9 expression following a 5-day drug washout period. While active acute suppression prevents immediate elastolysis, delayed or persistent matrix remodeling dynamics persist post-withdrawal, particularly under sub-optimal low-dose regimens. These findings underscore that while acute high-dose ASA is irreplaceable for arresting hyper-acute inflammatory tissue injury, careful post-withdrawal monitoring and tailored anti-platelet maintenance are essential to safeguard against subclinical arterial wall remodeling. Together, our results validate current clinical guidelines and offer a refined rationale for optimizing combination dosing strategies to prevent coronary artery aneurysms in pediatric patients.

## Data Availability

The original contributions presented in this study are included in the article. Further inquiries can be directed to the corresponding author.

## Abbreviations

The following abbreviations are used in this manuscript:

KD	Kawasaki disease
IVIG	Intravenous immunoglobulin
ASA	Aspirin
LCWE	Lactobacillus casei cell wall extract
MMP-9	Matrix metalloproteinance-9
NF-κB	Nuclear factor kappa-B
